# Evaluation of a genus-specific rGroEL_1-524_ IgM-ELISA and commercial ELISA kits during the course of leptospirosis in Thailand

**DOI:** 10.1038/s41598-021-99377-8

**Published:** 2021-10-05

**Authors:** Santi Maneewatchararangsri, Galayanee Doungchawee, Thareerat Kalambaheti, Viravarn Luvira, Ngamphol Soonthornworasiri, Pisut Vattanatham, Urai Chaisri, Poom Adisakwattana

**Affiliations:** 1 Department of Molecular Tropical Medicine and Genetics, Faculty of Tropical Medicine, Mahidol University, Bangkok, Thailand; 2 Center for Research and Innovation, Faculty of Medical Technology, Mahidol University Salaya Campus, Nakhon Pathom, Thailand; 3 Department of Microbiology and Immunology, Faculty of Tropical Medicine, Mahidol University, Bangkok, Thailand; 4 Department of Clinical Tropical Medicine, Faculty of Tropical Medicine, Mahidol University, Bangkok, Thailand; 5 Department of Tropical Hygiene, Faculty of Tropical Medicine, Mahidol University, Bangkok, Thailand; 6 Department of Tropical Pathology, Faculty of Tropical Medicine, Mahidol University, Bangkok, Thailand; 7 Department of Helminthology, Faculty of Tropical Medicine, Mahidol University, Bangkok, Thailand

**Keywords:** Biological techniques, Immunology, Health care

## Abstract

In the present study, we developed a genus-specific rGroEL_1-524_ IgM-ELISA assay for use in screening diagnosis of suspected leptospirosis among acute undifferentiated febrile illness patients during acute fever. The diagnostic accuracies of the rGroEL_1–524_ IgM-ELISA, commercial Panbio IgM-ELISA, and Virion-Serion Classic IgG-ELISA were evaluated using 133 Thai leptospirosis sera and 210 controls. Sensitivities were 91.7%, 59.6%, and 17.7% for acute infection, and the specificities were 92.6%, 90.2%, and 88.3% for the non-leptospirosis control, respectively. The rGroEL_1-524_ IgM-ELISA had high sensitivity, at 92.3% and 91.7%, among culture-positive and MAT-negative cases at 1–3 days post-onset of symptoms (DPO1–3), respectively. Impaired specificity on scrub typhus was found, possibly from antibody cross-reaction to ortholog GroEL. Commercial Panbio IgM-ELISA had sensitivities at DPO1–3 of 30.8% and 41.7% for culture-positive and MAT-negative cases whereas Virion-Serion IgG-ELISA showed sensitivities of 5.9% and 13.3%, respectively. The rGroEL_1-524_ IgM-ELISA could be useful as a screening test for early diagnosis. The performance of the commercial ELISA suggests the applicability of IgM-ELISA for diagnosis, while IgG-ELISA is useful for seroprevalence surveys. However, confirmation by reference tests is recommended.

## Introduction

Leptospirosis is recognized as a waterborne zoonosis with high incidence in tropical and sub-tropical areas, particularly rural areas and urban slum communities in developing and industrialized countries. Its epidemiology is undergoing changes due to global warming and migration. The disease continues to be a global public health burden, afflicting 0.1–1 per 100,000/population with a 10% case fatality rate annually; it also has a socio-economic impact^1^. Epidemic outbreaks occasionally occur and are associated with flooding in epidemiological settings including Thailand, the Philippines, New Caledonia, Hawaii, and Nicaragua. The Thai Bureau of Epidemiology, Department of Disease Control, Ministry of Public Health, reported incidence and case fatality rates in 2019 of 3.26 and 0.04 per 100,000/population, respectively, with demographic shifts in southern Ranong, Phang Nga, Yala, and northeastern Sisaket and Yasothon provinces. It is usually associated with farmers, laborers, students, and government services staff, and is also associated with recreational activities and travelers^2–4^. The disease is seasonal, with a peak incidence in the late rainy season to early winter, and occasionally occurs after high rainfall and flooding, such as in the epidemic outbreak in Loei in 2002^5,6^.

Humans are usually infected by pathogenic bacteria of the genus *Leptospira*, which are allocated into three distinct phylogenetic clusters by the virulence of the bacteria, comprising 13 pathogenic and 5 intermediately pathogenic species and free-living saprophytes^7^. The main disease-causing species in humans and other animals are pathogenic *Leptospira interrogans*, *L. borgpetersenii*, and *L. kirschneri*^8^; the intermediate group members that infect humans and mammals causing mild disease include *L. broomii, L. fainei*, *L. inadai, L. licerasiae*, and *L. wolffii*, whereas non-pathogenic species do not cause disease^7,9,10^. Based on serology, the *Leptospira* spp. are classified into more than 300 different serovars and clustered into at least 24 serogroups^8,11^. Human hosts commonly acquire infection by contact with bacterially contaminated urine, soil, or water through abraded skin or mucous membranes, which causes a wide range of clinical manifestations ranging from asymptomatic to mild or acute undifferentiated febrile illness (AUFI)^4^ to severe leptospirosis symptoms such as Weil’s disease, severe pulmonary hemorrhage syndrome, and aseptic meningitis, and is a potentially fatal illness^12,13^. Most patients present with non-specific febrile illness similar to other tropical diseases, such as dengue fever, rickettsioses, malaria, influenza, septicemic melioidosis, viral hemorrhagic fever, and enteric fever, making misdiagnosis possible^14–16^. Patients receiving early diagnosis and appropriate antibiotic therapies within 4–5 days post-onset of symptoms (DPO) have higher rates of recovery^17,18^. Delayed case diagnosis and late treatment can rapidly lead to severe complications where chemotherapy becomes useless^2,16^.

The World Health Organization suggests that the gold standard laboratory method for confirming leptospirosis diagnosis should be: (i) isolation of *Leptospira* by the culture method, (ii) detection of organism DNA by PCR, and (iii) detection of antibodies by microscopic agglutination test (MAT)^17^. MAT detects both immunoglobulin M (IgM) and IgG agglutinating antibodies. However, MAT provides low sensitivity at the early course of infection, as MAT can detect IgM antibodies after DPO 8 and requires paired-sera testing to confirm diagnosis. The test cannot differentiate current, recent, or past infections. Furthermore, MAT is technically demanding, time-consuming, and requires well-trained personnel for interpretation. Culturing provides definite proof of leptospiral infection and could identify locally pathogenic serovars. However, culture is not useful as a diagnostic tool because by the time a diagnosis is made by culture, antibodies are already detectable by serological techniques and the result is relatively delayed. MAT and culture methods have low diagnostic sensitivities but high specificity. Molecular diagnosis by PCR, quantitative PCR (*q*PCR), and recently whole genome sequencing are not affordable in primary healthcare and in rural areas with resource-limited settings^17,19^. Hence, the development of reliable and valid diagnostic tests providing high accuracy is needed for the diagnosis of leptospirosis, so that the disease can be diagnosed and treated early in its course.

Immunodiagnostics using the detection of IgM antibodies during acute illness, such as the enzyme-linked immunosorbent assay (IgM-ELISA), immunofluorescence assay (IFA), and immunochromatography (ICT) formats^20,21^, have been implemented for the diagnosis of infectious diseases in the tropics including leptospirosis, dengue fever, rickettsioses, and melioidosis^22–24^. IgM-ELISA is recommended by the World Health Organization (WHO) as useful in early diagnosis; the test has shown high sensitivity and specificity and is more sensitive than MAT. Commercially available ELISAs have shown inconsistent performance when evaluated in different epidemiological settings, and their accuracy requires systematic evaluation in Thailand^17,18,25^. The diagnostic accuracy of the commercial IgG-ELISA has been evaluated in limited studies and has not been evaluated in Thailand. The commercial Panbio IgM-ELISA has been evaluated in different settings, including Malaysia^26^, Hawaii^27^, Laos^28^, Southern Vietnam^29^, southern Sri Lanka^30^, and the French West Indies^31^. The Panbio IgM-ELISA demonstrated limited diagnostic sensitivity and specificity, at 76.1% and 82.6%, respectively, when evaluated in high-prevalence northeast Thailand^32^.

*Leptospira* infections cause upregulation of bacterial heat shock protein 60 (GroEL) in response to temperature stress conditions, eliciting long-lasting immune responses with high antibody titers. GroEL has shown to be a genus-specific immunodominant antigen, as revealed by anti-*Leptospira* immune serum and leptospirosis paired sera^33–35^. This protein has shown less cross-reactivity with sera from patients who have recovered from melioidosis or dengue hemorrhagic fever^33–35^. In this study, we developed an IgM-ELISA screening test using a genus-conserved region of GroEL_1-524_ (recombinant GroEL_1–524_) as antigen to detect specific IgM antibody in blood specimens of suspected leptospirosis cases among AUFI patients. We then evaluated its diagnostic performance for early leptospirosis diagnosis using local Thai blood samples as compared to the culture and MAT methods. We also assessed the usefulness of commercial ELISAs, Panbio *Leptospira* IgM-ELISA and the Virion-Serion classic *Leptospira* IgG-ELISA, as screening tests for detecting anti-leptospiral antibodies using Thai blood samples in the context of disease outbreaks and compared to *Leptospira* culture and MAT.

## Results

### Verification of GroEL_1-524_ sequence conservation within the genus ***Leptospira*** spp. and among orthologous GroEL proteins

The *Leptospira* GroEL_1-524_ sequence had a high degree of homology, at 99% (522/524) sequence identity to other leptospiral serovars in the *Leptospira* spp. To evaluate their protein-sequence conservation across genera, GroEL orthologs among other tropical infectious diseases including leptospirosis, scrub typhus, melioidosis, and malaria were examined. Orthologous GroEL proteins of influenza A and dengue viruses were not found in the NCBI database. The orthologous GroEL proteins of *L. interrogans, Burkholderia pseudomallei*, *Plasmodium vivax*, and *Orientia tsutsugamushi* organisms demonstrated 60.5%, 51.3%, and 51.9% identity, respectively, compared with the cloned *Leptospira* GroEL_1-524_ sequence. The GroEL_1-524_ sequence was highly conserved in the genus *Leptospira* spp. and shared conserved peptides among orthologous GroEL proteins.

### Prediction of GroEL_1-524_ linear B-epitope peptides

Two B-epitope peptides of 30-LGPKGRN-36 (85.7% identity) and 404-AAVEEGIVPG-413 (100% identity) have been shown to be highly conserved among leptospirosis, scrub typhus, melioidosis, and malaria pathogens. The epitope peptide similarity suggests a degree of immunological cross-reactivity among leptospirosis, scrub typhus, malaria, and melioidosis sera.

### Recombinant GroEL_1-524_ protein preparation

The C-terminal His-Tagged GroEL_1-524_ protein was produced as a soluble protein of estimated 58.7 kDa. The purified rGroEL_1–524_ protein was verified for antigenic specificity, which revealed a reactive band at 60 kDa (supplementary Fig. S1). The rGroEL_1–524_ protein was used as an antigen in the development of a recombinant antigen-based IgM-ELISA.

### IgM sera reactivity of leptospirosis and controls to rGroEL_1–524_ antigen by IgM-ELISA

**Figure 1:**
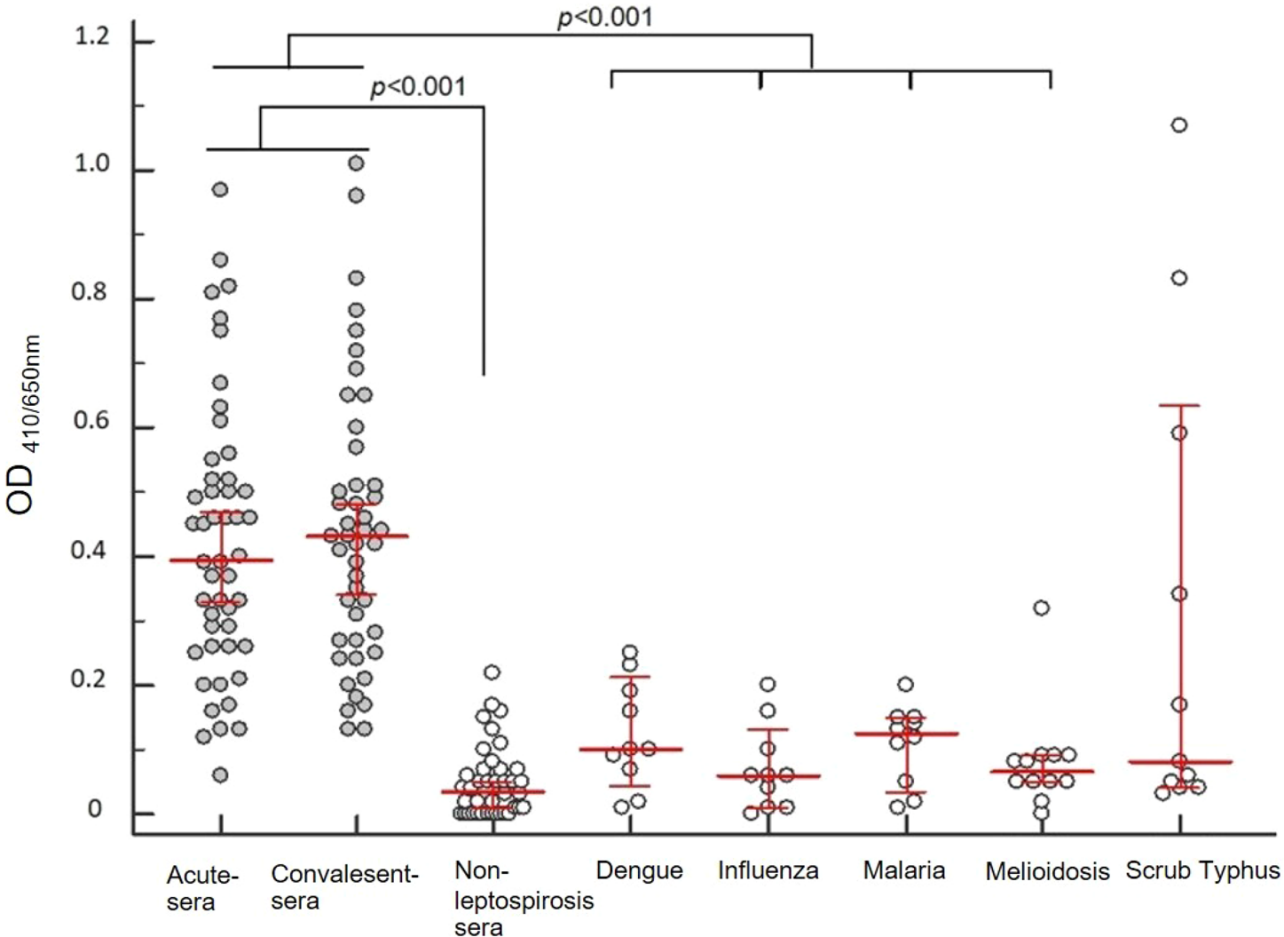
IgM reactivity of confirmed leptospirosis paired sera and controls assessed by rGroEL_1-524_ IgM-ELISA. The IgM reactivity of acute and convalescent leptospirosis sera (closed circle), non-endemic healthy and AUFI control plasma (open circle), and other febrile illness control samples (open circle), comprising dengue fever, influenza, malaria, melioidosis and scrub typhus subgroups, were assessed rGroEL_1-524_ IgM-ELISA assay. Individual IgM reactivity is expressed as AOD ELISA. The median AOD ELISA value and standard deviation (SD) of each subgroup are indicated. *P* < 0.001 is considered significantly different.

The IgM antibody reactivities of leptospirosis paired sera in an optimized rGroEL_1–524_ IgM-ELISA were presented as actual optical density (AOD) values with a range of 0–1.01. Median AOD values were 0.40 and 0.43 for leptospirosis paired sera, and were significantly higher than those of the non-leptospirosis control (*P* < 0.0001) and other febrile illness (*P* < 0.001). The IgM reactivities of leptospirosis sera versus scrub typhus sera were not significantly different (*P* = 0.15, *P* = 0.09, respectively) (Fig. 1). The median IgM reactivities of culture positive samples and seroconversion were 0.50 and 0.45 for acute sera and 0.25 and 0.34 for convalescent sera, respectively (Fig. 1).

### Determination of optimal cut-off values

To optimize cut-off values, receiver operating characteristic (ROC) curves were generated from leptospirosis paired sera and controls to define the optimal optical density (OD) of 0.15 to achieve an estimated sensitivity of 91.6% and 95.5% for the paired sera and a specificity of 87.5% for the controls.

The results of IgM detection by Panbio IgM-ELISA and IgG detection by Virion-Serion IgG-ELISA on leptospirosis sera and controls are illustrated with adjusted cut-off values in supplementary Fig. S2. The optimal threshold for Panbio IgM-ELISA cut-off value was ≥ 7 Panbio units, and the optimized cut-off value for Serion IgG-ELISA was ≥ 0.35 OD ELISA to achieve higher sensitivities.

### Diagnostic accuracy of rGroEL_1–524_ IgM-ELISA

Thai blood samples were randomly selected to evaluate rGroEL_1–524_ IgM-ELISA performance compared with reference methods. The diagnostic sensitivities were 91.7% for acute sera and 95.6% for convalescent sera (Table 1).

**Table 1:** Diagnostic performances of rGroEL_1-524_ IgM-ELISA and commercial IgM-ELISA (Panbio) and IgG-ELISA (Virion-Serion) during the course of leptospirosis.

Sera	rGroEL_1-524_ IgM-ELISA		Panbio IgM-ELISA^a^		Virion-Serion IgG-ELISA^b^	
	No. positive/total	Percent (95% CI)	No. positive/total	Percent (95% CI)	No. positive/total	Percent (95% CI)
**Sensitivity**						
Acute	44/48	91.7 80.0–97.7	31/52	59.6 45.1–72.9	9/51	17.7 8.4–30.9
Convalescence	43/45	95.6 84.9–99.5	30/42	71.4 55.4–84.3	23/47	48.9 34.1–63.9
**Specificity**						
Non-leptospirosis	7/44	92.6 82.1–97.9	5/51	90.2 78.5–96.7	5/43	88.3 74.9–96.1

^a^Optimized cut-off for Panbio IgM-ELISA was ≥ 7 panbiounits.

^b^Optimized cut-off for Virion-Serion IgG-ELISA was ≥ 0.35 OD ELISA.

The specificity was 92.6% for the non-leptospirosis control (Table 1), with corresponding AUC values of 0.93 (*P* < 0.001). Of the following febrile illness specificities, the results were 60.0% for dengue fever, 54.5% for scrub typhus, 81.8% for influenza, 70.0% for malaria, 91.7% for melioidosis, and 100% for other febrile illness whole blood samples. The diagnostic sensitivities of MAT-negative leptospirosis samples and culture-positive samples were 91.7% and 92.3% at DPO1–3, and 92.3% and 90.9% at DPO4–10, respectively (Table 2).

**Table 2:** Diagnostic sensitivities of rGroEL_1-524_ IgM-ELISA and Panbio *Leptospira* IgM-ELISA on MAT negative (≤ 1:50) acute samples of sero-conversion leptospirosis cases and culture-positive acute sera on DPO1-3, and DPO4-10.

Leptospirosis acute sera	rGroEL_1-524_ IgM-ELISA		Panbio IgM-ELISA^a^	
	No. positive/total	Percent (%)	No. positive/total	Percent (%)
**DPO1-3**				
MAT negative samples	11/12	91.7	5/12	41.7
Culture positive samples	12/13	92.3	4/13	30.8
**DPO4-10**				
MAT negative samples	12/13	92.3	3/10	30.0
Culture positive samples	10/11	90.9	2/11	18.2

^a^Optimized cut-off for Panbio IgM-ELISA was ≥ 7 panbiounits.

### Diagnostic performance of commercial ELISA test kits against local Thai sera

The performance of the commercial ELISAs was determined using the same panel samples of leptospirosis and non-leptospirosis sera. The diagnostic sensitivities of the Panbio IgM-ELISA were 59.6% for acute and 71.4% for convalescent sera, whereas the Virion-Serion IgG-ELISA showed 17.7% and 48.9% sensitivities for leptospirosis paired sera, respectively. The specificity was 88.3% for the non-leptospirosis control**.**

Sensitivities of the commercial Panbio IgM-ELISA were 41.7% for MAT-negative cases and 30.8% for culture-positive samples at DPO1–3 and 30.0% for MAT-negative cases and 18.2% for culture-positive samples at DPO4–10 (Table 2).

### Analysis of false negative results among leptospirosis samples from rGroEL_1–524_ IgM-ELISA

False negative results were found on seronegative (MAT ≤ 1:50) leptospirosis samples on paired-leptospirosis sera and culture positive samples; they had IgM reactivities of 0.06–0.13. Anti-IgM positive result of MAT-negative cases suggested insufficient GroEL-specific IgM antibody or levels under the limit of detection. False negative of culture-positive samples might have arisen from the window of active infection. (supplementary Table S3).

### Analysis of false-positive results among controls from rGroEL_1–524_ IgM-ELISA

IgM positivity among controls by rGroEL_1–524_ IgM-ELISA was 7.5% (supplementary Table S4). All false-positive samples were negative for anti-*Leptospira* IgM and IgG detection by commercial ELISA, suggesting that the false positives in the non-leptospirosis control may have arisen from non-specific binding or pre-existing antibodies (background antibody) in those samples.

The analysis of false-positive samples among the other febrile illness controls is summarized in supplementary Table S4. Melioidosis samples had 8.3% (1/12) false-positive results by rGroEL_1-524_ IgM-ELISA. Serum showed anti-*Leptospira* IgG positivity using the commercial test, which suggested antibody cross-reactivity from previous exposure. The scrub typhus sera showed IgM reactivity ranging from 0.03 to 1.07; 45.5% (5/11) were deemed false positives. Two of the false positives had anti-*Leptospira* IgM positivity, which suggested antibody cross-reactivity from recent or current exposure or leptospirosis-scrub typhus coinfection. Three of the false-positive results were likely caused by antigen cross-reactivity to ortholog GroEL antigen or pre-existing antibody from endemic leptospirosis.

Two of three false-positive malaria samples had anti-*Leptospira* IgG positivity by Virion-Serion IgG-ELISA detection. The false-positive reactions might be related to pre-existing antibodies from previous exposure. One malaria sample had false-positive result by an in-house IgM-ELISA, which suggested antigen cross-reactivity to ortholog GroEL antigen or pre-existing antibody.

Two of ten influenza samples had false-positive results by IgM-ELISA (AOD, 0.16–0.2) at 1:100 dilution, but all were negative for anti-*Leptospira* IgM and IgG detection by the commercial tests. False positives were likely caused by non-specific reactions. False positives of dengue samples were 40% (4/10) by in-house IgM-ELISA. Three of the false-positive samples tested negative for anti-*Leptospira* detection, which suggests that false positives may arise from endemic background antibodies. One false-positive dengue sample had anti-*Leptospira* IgM positivity, which suggested recent or current infection and leptospirosis-dengue coinfection.

## Discussion

Genus-specific antigen-based ELISAs using immunodominant outer membranes as antigens, such as LipL32, LipL41, Loa22, LigA, Lsa63, GroEL, and a combination (multiple antigens), have been widely developed for use as a screening test for leptospirosis^35–38^. The present study developed a prototype IgM-ELISA using a recombinant GroEL_1-524_ formatted antigen as an early laboratory screening test for leptospirosis and evaluated its diagnostic accuracy in the context of disease outbreaks in Thailand compared with reference methods. We produced rGroEL_1–524_ protein (C-terminal deletion of 22 amino acids) and used an ELISA antigen to detect anti-rGroEL_1–524_ IgM antibody during the course of illness for early diagnosis of suspected cases among AUFI caused by other infections. The heat shock GroEL chaperonin has shown as a diagnostic potential in leptospirosis based on its upregulated expression during infection (temperature upshift). GroEL has been shown to be an immunodominant antigen and has less cross-reactivity with melioidosis and dengue hemorrhagic fever^33–35^. The immunoreactivity of severe leptospirosis, such as pulmonary involvement and renal failure, to recombinant GroEL has been shown to have 90.6% sensitivity and 94.9% specificity^39^. In addition, the GroEL_1-524_ sequence is highly conserved within the genus *Leptospira* and shares lower sequence conservation with the orthologous GroEL. We evaluated the diagnostic performance of the rGroEL_1–524_ IgM-ELISA using leptospirosis paired-sera derived from northeastern Thailand, i.e., Loei, Nakhon Ratchasima, Sakol Nakhon, and controls from non-endemic Bangkok and other febrile illnesses, compared to MAT and culture methods. Leptospirosis sera from Loei were collected from an outbreak in 2002. Samples were found to be positive by *Leptospira* isolation (30 culture-positive acute sera) and by seroconversion criteria. A small sample size of 28 single MAT ≤ 1:400 leptospirosis sera was obtained.

In the present study, a single IgM-ELISA was designed as a highly sensitive screening test. The cut-off was determined to be 0.15 AOD for single IgM-ELISA testing to achieve an estimated sensitivity of 91.6% and 95.5% for paired sera and specificity of 92.5% and 76.0% among the non-leptospirosis and febrile controls, respectively. One limitation of acute-phase IgM testing with a single specimen is that people in endemic areas are expected to have pre-existing antibodies causing impaired specificity. ELISA results give no indication of the infecting serovar, and a confirmatory diagnosis of leptospirosis should be performed. IgM antibody usually persists for 5 months^40^, ELISA can be used as a simple and rapid laboratory screening test for the diagnosis of leptospirosis for several months after the onset of symptoms.

The sensitivities of the rGroEL_1-524_ IgM-ELISA were 91.7% and 95.6% for leptospirosis paired sera, and the specificity was 92.6% among the non-leptospirosis control. Lessa-Aquino et al.^35^ reported GroEL IgM-ELISA sensitivities of 90% and 92.0% and specificities of 53.8% and 62.5% in paired sera. A systematic review and meta-analysis of the performance of *Leptospira* IgM-ELISA averaged 84% sensitivity and 91% specificity for acute infection^41^. The rGroEL_1-524_ IgM-ELISA had higher diagnostic performance than previously reported^35,41^ due to the ability of the refined rGroEL_1-524_ molecule to encompass more antigenic moieties of the whole genus. The prototype IgM-ELISA had high sensitivities of 95.9% in culture-positive sera, 91.2% in seroconversion samples, and 88.2% in MAT-positive subgroups. With a cut-off of 0.2 AOD, expected test performance was 87.5% and 86.7% for sensitivity in paired sera and 81.5% specificity in the febrile control. The most prevalent serovars infecting patients in the sera used in this study were Bratislava, Autumnalis, Australis, New, Sarmin, and Bangkok^5^, while Autumnalis, Bratislava, and Pyrogenes were the most common serovars in Thailand in 2003–2012^42^. The in-house IgM-ELISA can detect IgM antibody as early as DPO1. The false negative results in the acute phase by in-house IgM-ELISA might be due to the long window by the dynamics of antibody production. Symptomatic patients may have no or low antibody levels at 1–2 weeks post-exposure, and the antibody titer will rise with time. We found two false negative convalescent sera, which might have been caused by a delayed response, which sometimes occurs over 30 days after infection^20^.

Leptospirosis infections are often under-reported due to false negatives among mild cases or those who have already received antibiotics, have suppressed immunity, or are in the very early or late phase of the immune response. In coinfection patients, weak or cross-reactions may occur. The false-positive rate among febrile patients is possibly caused by cross-reactivity, anti-GroEL_1-524_ IgM antibody from leptospirosis co-infections, or pre-existing IgM antibody in patients with recent exposure in endemic areas.

Commercial ELISA tests have been used for the diagnosis of leptospirosis in Thai endemic settings, including the Panbio *Leptospira* IgM-ELISA and Virion-Serion classic *Leptospira* IgM/IgG. The performance of commercial ELISA tests varies by geographical setting, with the sensitivity of the *Leptospira* IgM-ELISA being 35–76% and specificity being 76–98% in different endemic settings^27–32,41^. These ELISA tests use whole-cell lysates from pathogenic *L. interrogans*, intermediate *L. fainei*, or saprophytic *Leptospira biflexa* antigens to detect genus-specific anti-*Leptospira* IgM/IgG antibodies. Heterogeneous native antigens in ELISA tests may not recognize the local serovars, so their sensitivities are frequently poor and have been limited by the heterogeneity of host immunological responses to native antigens.

Whole cell-based ELISAs (Panbio IgM- and Virion-Serion IgG-ELISA) demonstrated poor sensitivity against local Thai leptospirosis paired sera in the present study (Table 1). Another study found that the Panbio IgM-ELISA showed 90.8% positivity among samples from northeastern Thailand^32^. IgM antibodies appear earlier than IgG antibodies and remain detectable at low titers for months or even years. An IgG titer of 1:100 can be present due to past infection. Whole cell-based ELISA, which is affected by serogroup-specific antigens or whole-cell antigens, has poor sensitivity and may not recognize local infectious strains in different endemic areas^20,32,43^. IgM-dominant and IgG-dominant *L. biflexa* serovar Patoc antigen, i.e., LPS, cytoplasmic, secreted, and envelop membrane proteins, do not encompass local infecting serovars in the genus *Leptospira. L. biflexa* Patoc I antigen is known to cross-react with several serovars, but usually does not cross-react with animal strains. The most predominant infecting serovars in suspected patients from 2003–2004 in Thailand were Autumnalis, Bataviae, Pyrogenes, Javanica, Hebdomadis and Grippotyphosa^44^. The most predominant infecting serovars between 2010 and 2015 were serovars (associated reservoir) Shermani (cattle, buffalo, pig), Bratislava (livestock, i.e., cattle, buffalo), Panama, and Sejroe (rodents)^45^. Another study examined the potential risk of a leptospirosis outbreak in Bangkok and Nakhon Pathom between 2011 and 2012, and found *L. wolffii* and intermediate *L. licerasiae*^46^. Poor sensitivity can be attributed to several factors, such as acute serum being collected too early in the course of illness (less than DPO4–5), inadequate IgM antibody levels in the patient, second or subsequent episode of infection leading to IgG antibody production, and patient receiving antibiotic medication. To improve specificity due to high background antibodies among the seropositive population requires validation and adjustment of the cut-off. In this study, we optimized cut-offs for commercial ELISAs. An adjusted Panbio unit of ≤ 7 showed a sensitivity improvement to 54.8% for the DPO1–3 acute phase and specificity of 86.6%. The Panbio IgM-ELISA provided sensitivity and the ability to detect IgM antibodies as early as DPO1–3. Virion-Serion IgG-ELISA with an adjusted cut-off provided 17.7% and 48.9% sensitivity on leptospirosis paired sera and 81.7% specificity among the controls. False-positive IgG detection was 19% among the controls (10% for non-endemic samples and 25% for each infection, i.e., dengue, malaria, scrub typhus, and melioidosis). IgG seropositivity rates of 17.7% and 48.9% for paired sera suggest that IgG responses should be due to epidemic leptospirosis in Loei rather than background antibody, with 10% seropositivity among healthy and AUFI patients in low-prevalent leptospirosis areas such as Bangkok.

Several studies have reported that ELISA-based assays detect anti-*Leptospira* IgM antibodies earlier than MAT assay during the early course of disease^20,47,48^. Nicofa et al.^20^ suggested that *Leptospira*-specific IgM antibodies appear 1–2 days earlier than the agglutinating antibodies detected in the MAT assay; therefore, earlier positive results could be expected from our genus-specific IgM detection. We found that the sensitivities in seronegative and culture-positive acute sera were 91.2%, and 95.9% for the prototype IgM-ELISA and 63.2% and 50.0% for commercial IgM-ELISA, respectively. An unvalidated diagnostic test with poor specificity may contribute to overdiagnosis of leptospirosis, because IgM antibodies from past infections are frequently detected among people living in endemic areas^20,48^.

The cross-reactivity of the rGroEL_1-524_ IgM-ELISA was evaluated using sera from the local population and a non-leptospirosis febrile control group. The specificity of anti-*Leptospira* IgM detection is limited in pathogens expressing orthologous GroEL proteins, such as scrub typhus, malaria, and melioidosis, causing IgM cross-reactivity in the rGroEL_1-524_ IgM-ELISA. High anti-*Leptospira* IgM levels in sera collected from patients along the Thai-Myanmar border have been reported^49,50^. However, it should be noted that cross-reactivity with bacterial infections can occur when patients harbor co-infections or have cross-reactive antibodies, especially in the early phase of leptospirosis when the IgM-ELISA lacks full specificity^20^.

The varied sensitivities likely reflect different case definitions and control groups, timing of collection, local prevalent serovar distribution, and the platform and protocol used in detection. A significant limitation of the rGroEL_1-524_ IgM-ELISA was poor specificity for leptospirosis-endemic areas. The test specificity was affected by ortholog GroEL antigen cross-reactivity, antibody cross-reactivities caused by previous exposure, and co-infections. Co-infections with leptospirosis were not assessed in samples from other febrile illnesses. The use of the rGroEL_1-524_ IgM-ELISA as a screening test for leptospiral infection would facilitate the difficult reference and differential tests. However, the test should not be used as the sole criterion for diagnosing leptospirosis. The ELISA results must be confirmed by convalescent serum. MAT is still recommended for disease confirmation and epidemiological study, and *Leptospira* isolation and molecular characterization should be performed for confirmation of the infecting serovars^51^.

## Conclusion

Our data demonstrated that IgM-ELISA using rGroEL_1-524_ antigen has sufficiently high sensitivity to screen for anti-GroEL_1-524_ IgM antibodies in the early leptospirosis diagnosis of suspected cases and among high-risk groups during leptospirosis epidemics. However, diagnostic specificity needs to be improved for implementation in areas with high levels of infectious tropical diseases. The commercial ELISA performance data suggest the applicability of IgM-ELISA for early diagnosis during disease outbreaks in low-prevalence areas for leptospirosis. IgG-ELISA is useful for seroprevalence surveys; however, confirmation by reference tests is recommended.

## Methods

### Ethics and biosafety

The protocol for using achieved sera and patient data was performed in concordance with the recommendation of the Declaration of Helsinki. Documentary Proof of Exemption Review was obtained from the Ethics Committee of the Faculty of Tropical Medicine, Mahidol University (MUTM-EXMPT2017-005). The written informed consents were obtained from participants. Sample anonymity was maintained, and all samples were re-coded without name and hospital ID. Biosafety was approved by the institute’s Biosafety Committee (MU2019-002).

### Study design

A retrospective study was carried out to assess the diagnostic performance of rGroEL_1–524_ IgM-ELISA and to evaluate commercial whole-cell antigen-based ELISA performance using local Thai blood samples compared with culture and MAT methods. The laboratory investigations were conducted at the Faculty of Tropical Medicine, Mahidol University, in Bangkok.

### Reference leptospirosis diagnosis

Patients’ specimens were investigated by cultivation or MAT. The reference diagnosis was conducted at Loei Provincial Hospital. *Leptospira* isolation was performed on the day of patient hospitalization by culturing blood specimens in EMJH (Ellinghausen-McCullough-Jonson-Harris) medium, followed by incubation for 16 weeks^5^. Sera were tested by MAT assay with 20 reference *Leptospira* serovars, as described previously^5,36^. Single leptospirosis sera were confirmed for MAT titers at the Faculty of Tropical Medicine, Mahidol University. MAT-positive criteria were defined as single MAT titer of ≥ 1:400 in a single specimen, sero-conversion from negative to titer ≥ 1:400, or  a fourfold rise in MAT titer using paired sera. A MAT-negative sample was defined as MAT titer ≤ 1:50^17,21,32,47,52^.

### Leptospirosis patients and sera

A suspected leptospirosis case was clinically diagnosed based on WHO criteria, i.e., AUFI in patients (fever ≥ 38 °C) with headache and myalgia and a history of exposure to animal reservoirs or flooded environments^17^. A confirmed leptospirosis case is defined as a clinically diagnosed, suspected leptospirosis case combined with positive laboratory diagnosis by the culture method or MAT assay.

**Figure 2:**
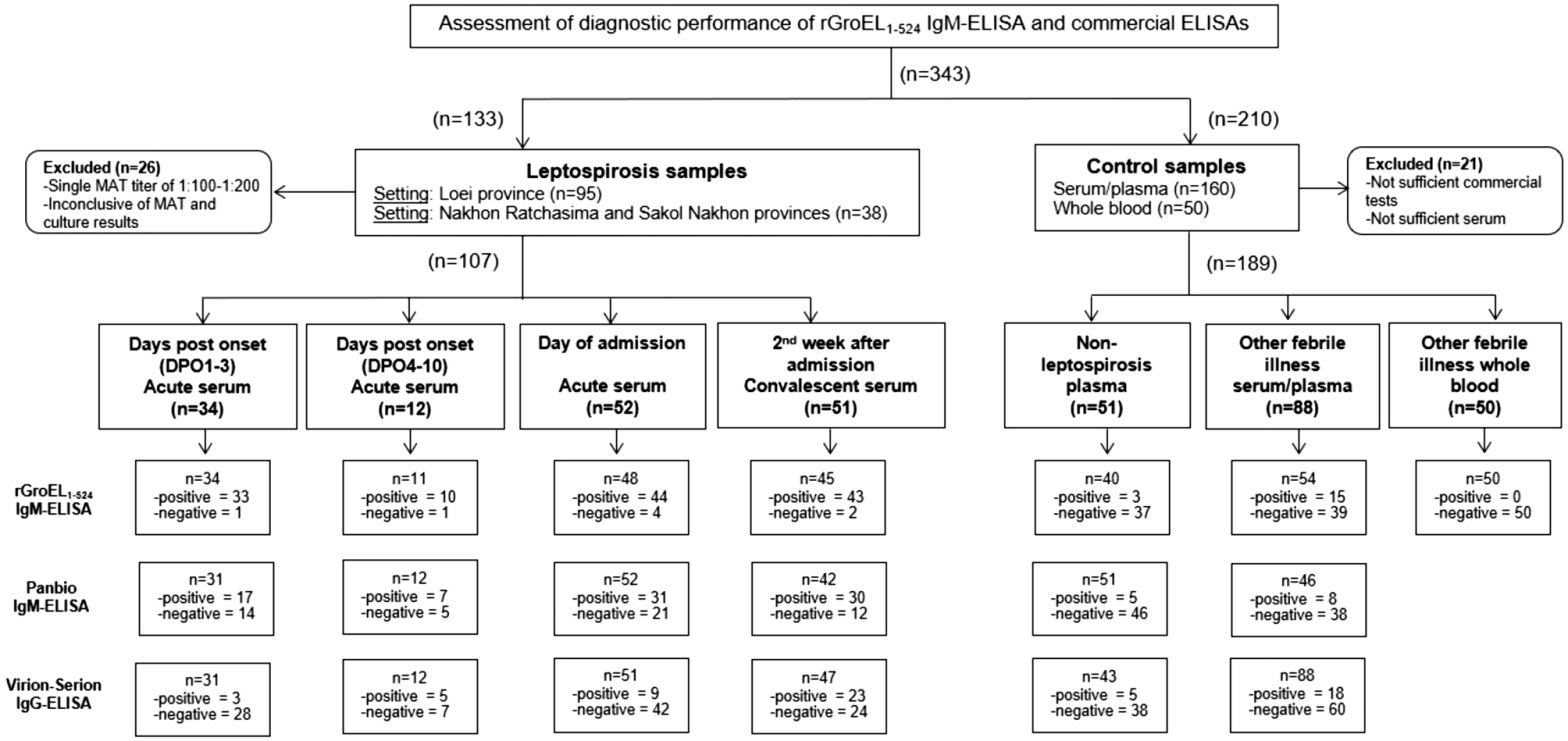
Flow diagram of the assessment of diagnostic accuracy of rGroEL_1-524_ IgM-ELISA, and commercial ELISAs. Confirmed leptospirosis sera (n = 133) and control samples (n = 210) were subjected to an assessment of the diagnostic sensitivity and specificity of the following tests: (i) rGroEL_1–524_ IgM-ELISA, and commercial (ii) Panbio *Leptospira* IgM-ELISA, and (iii) Serion-Virion classic *Leptospira* IgG-ELISA. Leptospirosis sera were acute sera collected on the date of admission (n = 52) and convalescent-sera collected in the later 2 weeks (n = 51). Acute-phase sera were classified according to days post-onset of symptoms (DPO) into DPO1-3 (n = 34), and DPO4-10 (n = 12) acute serum. Control samples were 51 non-leptospirosis plasma (seronegative and negative for leptospirosis IgM detection), 88 serum or plasma samples and 50 whole blood of laboratory-confirmed infectious diseases other than leptospirosis. Of 343 sera, 26 leptospirosis sera and 21 control samples were excluded from study. Positive results of the commercial ELISAs were considered using the recommended cut-offs.

Leptospirosis sera (n = 133) were obtained from patients during an epidemic outbreak at Loei Provincial Hospital (n = 95) and sporadic cases in the Nakhon Ratchasima and Sakhon Nakhon provinces (n = 38)^5,36^ (Fig. 2). Leptospirosis sera (n = 133) were acute sera (n = 52) and classified as DPO1–3 (n = 34), DPO 4–10 (n = 12), and convalescent sera (n = 51). Among the sera, samples with a single MAT titer of 1:100–1:200 (n = 26) were excluded. The most prevalent serogroups (serovars) among the MAT-positive sera were Autumnalis (Autumnalis, New), Australis (Australis, Bangkok, Bratislava), Icterohaemorrhagiae (Copenhageni), Sarmin (Sarmin), and Sejroe (Sejroe)^5^.

### Control samples

To assess the specificity of the ELISA tests, a panel of control samples (n = 210) consisting of 60 non-leptospirosis plasma (seronegative and negative for leptospirosis IgM detection) and 150 laboratory-confirmed infectious diseases other than leptospirosis were used (Fig. 2). Non-leptospirosis control were 60 plasma were collected from healthy volunteers and febrile plasma (n = 60) at the Hospital for Tropical Diseases, Bangkok in 2014. Other laboratory-confirmed febrile illnesses included seropositive dengue paired sera (n = 20) collected from classic dengue fever patients at Sisaket Provincial Hospital, Srisaket Province in 2013; scrub typhus samples (n = 20) that were PCR positive and sero-positive acute serum collected at Umphang Hospital, Tak Province in 2018; influenza paired sera (n = 20) collected from HI seroconversion of H1N1-infected patients; malaria plasma (n = 20) collected from malaria *vivax*-positive patients in Tak Province (n = 20); and melioidosis sera (n = 20) collected from IgM-seropositive melioidosis patients from northeastern Thailand before 2018. Whole blood samples (n = 50) were collected from AUFI patients admitted to the hospital for Tropical Diseases, Bangkok, from 2013 to 2015. The samples included laboratory-confirmed murine typhus (n = 15), dengue (n = 30), and bacterial sepsis (n = 5) caused by *E. coli*, *Streptococcus agalactiae*, *Salmonella* Typhi, and Viridans Streptococci infections (Fig. 2)^14^. The samples were collected in microtubes and stored at − 70 °C.

### Production of a transformed *E. coli* carrying a recombinant *GroEL*_*1–524*_–pET23a(+) plasmid

Briefly, a DNA sequence encoding for GroEL_1-524_ was amplified from genomic DNA extracted from *L. interrogans* serovar Icterohaemorrhagiae by PCR reaction using specific primers *GroEL*-*Nde*I/F (5ʹ-GGCCCATATGGCGAAAGATATTGAATAT-3ʹ) and *GroEL*-*BamH*I/R (5ʹ-TTGGATCC ATCTGGTTTGTCTGTGATTGT-3ʹ). Amplification was performed according to the following conditions: one cycle of 94 °C for 5 min, 29 cycles of 94 °C for 1 min, 55 °C for 1 min, and 72 °C for 1.30 min, followed by a final period of 72 °C for 5 min. The PCR product was analyzed under 1% agarose gel electrophoresis and visualized by gel documentation (Bio-Rad, California, USA).

The *GroEL*_*1-524*_ fragment was digested with *Nde*I and *Bam*HI restriction endonucleases and ligated into a linearized plasmid backbone to produce a recombinant *GroEL*_*1****–****524*_-pET23a(+) plasmid, which was subsequently introduced into BL21(DE3) *E. coli*. The *GroEL*_*1****–****524*_ sequence was verified by standard sequencing (Bioneer, Daejeon, Republic of Korea). The genetic map of the *GroEL*_*1–524*_-pET23a(+) plasmid is illustrated in supplementary Fig. S1.

### Production of recombinant GroEL_1–524_ protein

Recombinant GroEL_1–524_ protein was produced under an *E. coli* expression system using a transformed *E. coli* strain bearing the recombinant *GroEL*_*1*–*524*_ plasmid. Briefly, *E. coli* was grown in Luria–Bertani broth containing 100 µg/mL ampicillin at 37 °C with 200 rpm shaking until the culture reached an OD_600nm_ of 0.5. Thereafter, isopropyl β-d-1-thiogalactopyranoside (IPTG, 1 mM) was added to induce rGroEL_1-524_ protein expression at 37 °C with 200 rpm shaking for 3 h.

A soluble fraction from the IPTG-induced bacteria containing rGroEL_1-524_ protein was prepared in phosphate-buffered saline (1× PBS, pH 7.4) using a French pressure cell press at 30 kilo-pounds per square inch, repeated four times. The rGroEL_1-524_ protein was purified from the soluble proteins by native affinity chromatography using Ni^2+^-sepharose (GE Healthcare, Uppsala, Sweden). The purified rGroEL_1-524_ protein was concentrated in 1× PBS (pH 7.4) using a 3-kDa cut-off Amicon Ultra filter (Merck Millipore, MA, USA), and the protein concentration was determined using Bradford assay (Thermo Fisher Scientific, MA, USA). Aliquots of the protein (1 mg/mL) were lyophilized using the Labcono Freeze Dry system and then kept at − 70 °C.

### SDS-PAGE and Western blotting

Protein was analyzed under 13% SDS-PAGE gel electrophoresis, denaturing conditions, and Coomassie Brilliant Blue G250 stain. Antigenic specificity testing of the rGroEL_1-524_ protein was performed by probing the blotted membrane with anti-6× His-Tag monoclonal antibody (1:1000) (R&D Systems, MN, USA) for 1 h at 25 °C, followed by HRP-conjugated goat anti-mouse IgG secondary antibody (1:2,000) (Jackson ImmunoResearch, PA, USA) for 1 h at 25 °C (Southern Biotechnology, AL, USA). The reactive band was developed using 3,3-diaminobenzidine (DAB) chromogenic substrate (Thermo Fisher Scientific, MA, USA).

### In-house rGroEL_1–524_ IgM-ELISA

Recombinant GroEL_1–524_ (1 μg) immobilized ELISA strips (Jet Biofil, Guangzhou, China) were prepared as follows: rGroEL_1–524_ protein in 100 µL of carbonate–bicarbonate buffer (pH 9.6) was immobilized on ELISA wells at 37 °C for 24 h, and the antigen-coated wells were washed using washing buffer (300 µL/well of PBST; 0.05% Tween 20 in 1× PBS, pH 7.4). Washing was conducted by an automated microplate washer (Tecan Trading AG, Switzerland) three times to remove unbound material. The coated wells were then incubated with blocking reagent (300 µL of 1% BSA in 1× PBS) for 1 h at 37 °C, followed by incubation of the pre-blocked wells with 2% sucrose solution (300 µL) at 25 °C for 1 h. The ELISA wells were washed after each incubation step, as described above, and then air-dried. The pre-blocked rGroEL_1–524_ ELISA strips were packed with desiccant in press-seal bags and stored at − 20 °C until use.

To detect anti-GroEL_1-524_ IgM antibody, serum dilution (1:100, 100 µL) in a serum diluent (1× PBS containing 0.2% gelatin, 0.2% BSA), along with an internal positive control (pooled MAT-positive patient sera, where the adjusted AOD exceeded 0.2) and a reagent control (serum diluent) were incubated in pre-blocked antigen-coated wells at 37 °C for 1 h, followed by washing three times with PBST. Thereafter, HRP-conjugated goat anti-human IgM antibody (100 μL, 1:2,000) (Southern Biotechnology, AL, USA) was added to ELISA wells at 37 °C for 1 h incubation. ABTS chromophore diammonium salt (EMD Millipore, Germany) substrate solution (1 mg/mL ABTS tablet in 0.1 M sodium citrate buffer) was added (100 µL), and the plate was incubated for 15 min at 37 °C, after which 1% SDS solution (100 µL) was added to stop the reaction. The OD was measured at a wavelength of 410 nm against the reference at 650 nm (OD_410nm/650 nm_) using a microplate reader (Bio-Tek Instruments, VT, USA). Sample AOD was calculated by subtracting the OD of the reagent blank. The IgM-ELISA assay is valid when the OD of the reagent blank is < 0.2 and the positive AOD control is ≥ 0.2. A rGroEL_1-524_ IgM-ELISA protocol was optimized, and the optimal concentration of rGroEL_1-524_ was 1 µg/well; serum dilution was 1:100 and secondary antibody dilution was 1:1000–1:3000 dilutions.

### Panbio *Leptospira* IgM-ELISA

The diagnostic performance of the commercial Panbio *Leptospira* IgM-ELISA (Abbott Diagnostics, Illinois, USA) (Lot no. 02P10E001), using *Leptospira* genus-specific antigen, was assessed in Thai blood samples. The Panbio IgM-ELISA protocol was performed per the manufacturer’s instructions, measuring absorbance at OD_450nm/650 nm._ An index value was calculated by dividing the sample absorbance by the cut-off value. The result was expressed as Panbio units (index value multiplied by 10). Interpretation of the validity results was as follows: Panbio units (anti-*Leptospira* IgM) < 9 was a negative result, suggesting no evidence of recent infection, Panbio units ≥ 9 to < 11 was an equivocal result, suggesting possible recent infection, and Panbio units ≥ 11 was positive by IgM detection and interpreted as a recent or current infection. An equivocal result was considered a positive result. The Panbio IgM-ELISA test performance showed 96.5% sensitivity and 98.5% specificity and has been validated to detect *Leptospira* infections by serovars Pomona, Copenhageni, Australis, Canicola, Grippotyphosa, Tarsassovi, Hardjo, Madanesis, Kremastos, Nokolaevo, Cellodoni, Szwajizak, and Djasiman.

### Virion-serion classic *Leptospira* IgG-ELISA

Institute Virion-Serion ELISA Classic *Leptospira* IgG (Institut Virion/Serion GmbH, Warburg, Germany) (order no. ESR 125 G) was used to detect anti-*Leptospira* IgG antibody from serum or plasma using a crude membrane extract of *L. biflexa* serovar Patoc strain Patoc I, which contains genus-specific epitopes for all *Leptospira* spp. The Virion-Serion IgG-ELISA procedure was performed per the manufacturer’s instructions, with absorbance measured at OD_405nm/650 nm._ To interpret the qualitative results, the upper and lower cut-off range was calculated according to parameters provided with the kit. Actual OD (AOD) value (anti-*Leptospira* IgG) lower than the cut-off was considered a negative result suggesting no evidence of past exposure, an AOD value in the cut-off range was a borderline result suggesting possible past exposure, and an AOD value higher than the upper cut-off was positive by IgG detection, suggesting previous exposure. A borderline value was considered a positive result. The diagnostic performance of the Virion-Serion *Leptospira* IgG-ELISA was 96.7% sensitivity and 99.8% specificity.

### Evaluation of diagnostic accuracy

The Standards for Reporting of Diagnostic Accuracy studies (STARD 2015) checklist for reporting diagnostic accuracy is provided in supplementary Table S6.

Sample size was estimated as a minimum of 35 cases and control samples to achieve 90% sensitivity and specificity at a 95% confidence interval (CI) and 7% precision. All sera were tested as anonymous samples. Leptospirosis sera and controls (Fig. 1) were randomly selected to evaluate the performance of the following tests: (i) rGroEL_1–524_ IgM-ELISA, (ii) commercial Panbio *Leptospira* IgM-ELISA, and (iii) Virion-Serion Classic IgG-ELISA. The estimated diagnostic sensitivity and specificity with 95% CI were calculated by a 2 × 2 cross-tabulation table.

### Bioinformatics

Conservation of selected GroEL sequences in the genus *Leptospira* and among GroEL orthologs was determined using Clustal Omega multiple sequence alignment program interface^53^, and the results were analyzed using the BioEdit sequence alignment editor tool. Linear B-cell epitopes of the *L. interrogans* serovar Icterohemorrhagiae GroEL sequence were computationally predicted using a Bepipred-1.0 Linear Epitope Prediction tool^54^

### Statistical calculations

Data were collected in Microsoft Excel and analyzed using MedCalc Statistical Software version 19.2.5 (MedCalc Software Ltd, Ostend, Belgium; https://www.medcalc.org; 2020). Diagnostic parameters were calculated as follows: sensitivity = [(true positive (TP)/(TP + false negative (FN))] × 100; specificity = [(true negative (TN))/(TN + false positive (FP))] × 100. Normal distribution was tested using the Kolmogorov-Smirnov test. The Mann-Whitney test was used in non-normal distributed data. *P* < 0.05 was considered statistically significant.

## Supplementary Information

Supplementary Information.

## Data Availability

The datasets used and analyzed from the current study are available from the corresponding author on reasonable request.
