# Supplementary material for: Evaluation of a genus-specific rGroEL1-524 IgM-ELISA and commercial ELISA kits during the course of leptospirosis in Thailand
Source: Sci Rep. 2021 Oct 5;11:19785. doi: 10.1038/s41598-021-99377-8 (PMC8492722; doi:10.1038/s41598-021-99377-8)
Supplement: Supplementary file 1 — Supplementary Information. [file 41598_2021_99377_MOESM1_ESM.doc]

**Supplementary Information**

**Title of the manuscript: Evaluation of a genus-specific rGroEL_1-524_ IgM-ELISA and commercial ELISA kits during the course of leptospirosis in Thailand**

**Author List:** Santi Maneewatchararangsri, Galayanee Doungchawee, Thareerat Kalambaheti, Viravarn Luvira, Ngamphol Soonthornworasiri, Pisut Vattanatham,

Urai Chaisri, Poom Adisakwattana.


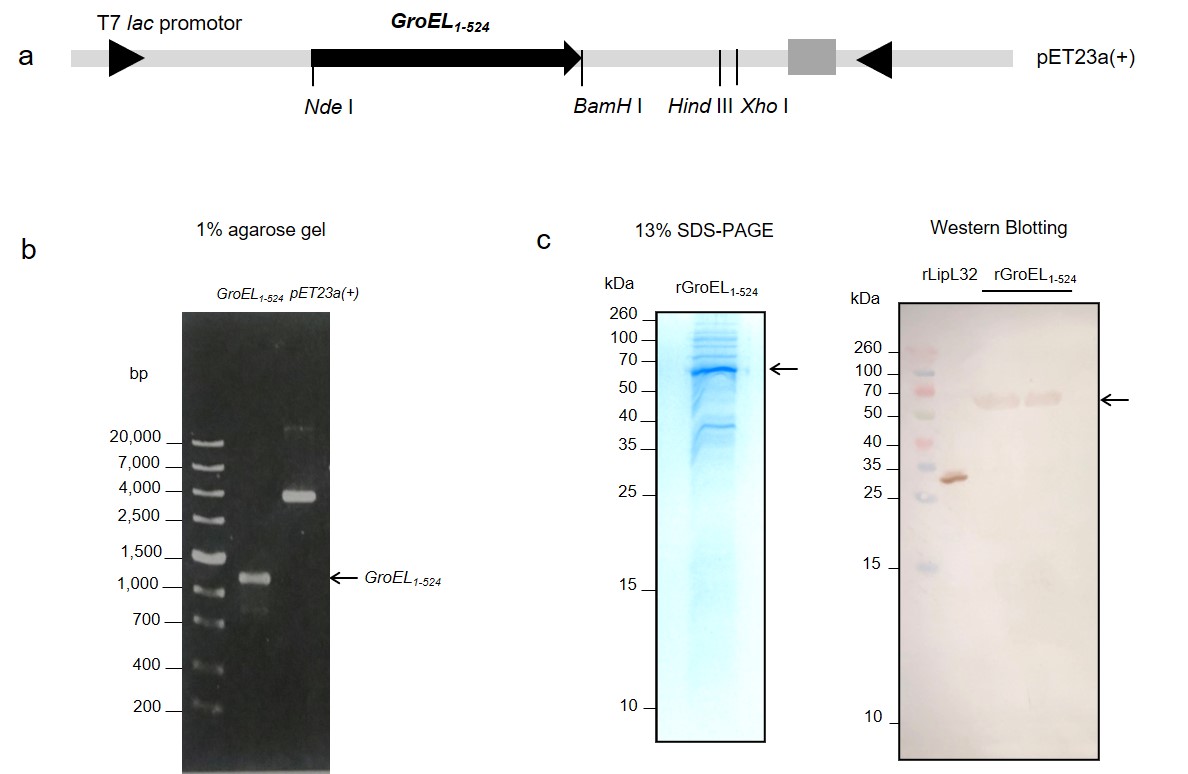


**Supplementary Fig. S1.** Production of recombinant GroEL_1-524_ antigen.

Genetic map of *GroEL_1-524_* DNA fragment inserted into pET23a (+) plasmid *via Nde*I and *BamH*I restriction sites (A). PCR amplicon of *GroEL_1-524_* at the calculated 1,338 bp and *pET23a(+)* plasmid DNA were analysed under 1% agarose gel (B). Recombinant GroEL_1-524_ protein (C-terminal deletion of 22 amino acids) was analyzed for molecular size at calculated 58.7 kDa under 13% SDS-PAGE gel (C) and the protein was verified for antigenic specificity by showing a reactive band at the relative size of 58.7 kDa (C).


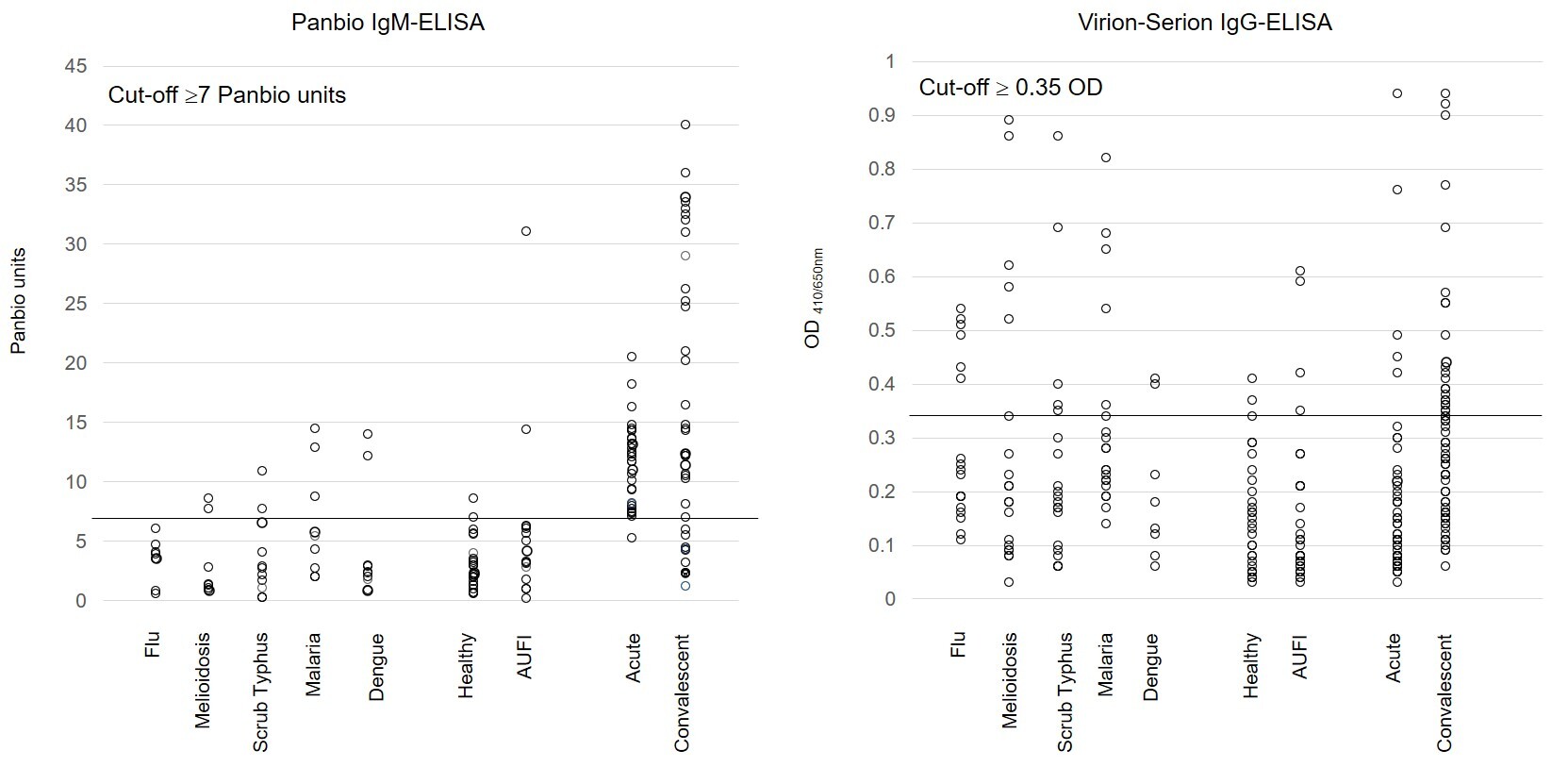


**Supplementary Fig. S2.** IgM and IgG reactivities on leptospirosis paired sera, and controls.

IgM reactivity was assessed by Panbio *Leptospira* IgM-ELISA and represented as Panbiounits and the IgG reactivity was assessed by Virion-Serion classic *Leptospira* IgG-ELISA and represented as sera absorbance (OD ELISA). Optimized cut-off values for each ELISA test are indicated.

**Supplementary Table S1.** IgM sera reactivity and diagnostic performance of the GroEL_1-524_ IgM-ELISA assessed by leptospirosis paired sera and controls and compared to culture and/or MAT reference standard.

| **Sera** | **Reactivity^1^** | **Positive /total** | **Sensitivity**  **(95% CI)** | **AUC** |
| --- | --- | --- | --- | --- |
| **Leptospirosis ~~paired~~ sera** |  |  |  |  |
| Leptospirosis | 0.39 (0.06-1.01) | 87/93 | 93.5% (86.5-97.6) | 0.93 |
| MAT≥1:400 | 0.47 (0.13-0.78) | 15/17 | 88.2% (66.8-98.6) | 0.91 |
| Seroconversion | 0.45 (0.13-1.01) | 52/57 | 91.2% (85.4-99.9) | 0.93 |
| Culture positive | 0.37 (0.06-0.92) | 47/49 | 95.9% (85.2-99.5) | 0.94 |
| **Controls** |  |  |  |  |
| Non-leptospirosis | 0.03 (0-0.22) | 37/40 | 92.5% (79.6-98.4) | 0.93 |
| Other febrile illness | 0.08 (0-1.07) | 39/54 | 72.2% (58.4-83.5) | 0.83 |
| Dengue | 0.10 (0.01-0.25) | 6/10 | 60.0% (26.2-87.8) | 0.93 |
| Scrub Typhus | 0.30 (0.03-1.0) | 6/11 | 54.5% (23.3-83.2) | 0.88 |
| Influenza | 0.06 (0-0.2) | 9/11 | 81.8% (48.2-97.7) | 0.95 |
| Malaria | 0.13 (0.02-0.2) | 7/10 | 70.0% (34.7-93.3) | 0.94 |
| Melioidosis | 0.06 (0-0.32) | 11/12 | 91.7% (61.5-99.7) | 0.97 |
| Other febrile illness whole blood | 0.02 (0-0.1) | 50/50 | 100% (92.8-100) | 0.99 |

Sensitivity was calculated from no. positive/total samples. Specificity was calculated from no. negative/total samples. Other infectious diseases consisted of melioidosis (n=12), scrub typhus (n=11), influenza (n=11), malaria (n=10), and dengue (n=10).

^1^ Median OD value (minimum-maximun OD values)

**Supplementary Table S2. Diagnostic performances of commercial IgM-ELISA (Panbio) and IgG-ELISA (Virion-Serion) using recommended cut-offs and the optimized cut-offs and culture and/or MAT as reference standard during the course of leptospirosis.**

| **Sera** | **Panbio IgM-ELISA** | | | | **Virion-Serion IgG-ELISA** | | | |
| --- | --- | --- | --- | --- | --- | --- | --- | --- |
|  | **Recommended cut-off**  **(≥9 Panbio units)** | | **Adjusted**  **cut-off^2^**  **(≥7 Panbio units)** | | **Recommended cut-off**  **range** | | **Adjusted**  **cut-off^3^**  **(≥0.35 AOD)** | |
|  | **No. ^1^** | **(%)** | **No.** | **(%)** | **No.** | **(%)** | **No.** | **(%)** |
| Sensitivity |  |  |  |  |  |  |  |  |
| Acute | 23/52 | 44.2 | 31/52 | 59.6 | 4/51 | 7.8 | 9/51 | 17.7 |
| Convalescence | 28/42 | 66.7 | 30/42 | 71.4 | 12/68 | 17.6 | 23/87 | 48.9 |
| Specificity |  |  |  |  |  |  |  |  |
| Non-leptospirosis | 39/41 | 95.1 | 46/51 | 90.2 | 60/60 | 100 | 38/43 | 88.3 |

Sensitivity was calculated from no. positive/total samples. Specificity was calculated from no. negative/total samples.

^2^ Optimized cut-off for Panbio IgM-ELISA was ≥7 panbiounits. ^3^ Optimized cut-off for Virion-Serion IgG-ELISA was ≥0.35 OD ELISA.

**Supplementary Table S3.** Analysis of false negative results of leptospirosis sera from rGreoEL_1-524_ IgM-ELISA.

| **Leptospirosis sera (DPO)** | **No.** | **Culture** | **MAT**  **titer** | **Panbio IgM-ELISA** | **Virion-Serion IgG-ELISA** | **Analysis of false negative results** |
| --- | --- | --- | --- | --- | --- | --- |
| Acute | A14 | Nd. | ≤1:50 | Positive | Negative | -Window period^1^  -Insufficient IgM antibody^2^ |
| DPO3 | A38 | Positive | 1:200 | Negative | Negative | -Active infection^3^  -Window period |
| DPO4 | A43 | Positive | 1:200 | Negative | Negative | -Active infection  -Window period |
| Acute | A45 | Nd. | 1:800 | Positive | Negative | -Insufficient IgM antibody |
| Convalescence | B25 | Positive | ≤1:50 | Positive | Negative | -Active infection  -Window period  -Insufficient IgM antibody |
| Convalescence | B42 | Positive | Nd. | Positive | Negative | -Active infection  -Window period  -Insufficient IgM antibody |

Nd., not determined.

DPO, days post-onset of symptoms.

^1^ blood collection too early.

^2^ Insufficient anti-GroEL_1-524_ antigen-specific IgM antibody in blood samples.

^3^ Active infection: defined as a positive *Leptospira* culture sample.

**Supplementary Table S4.** **Analysis of false-positive samples among the other febrile illness control samples from rGroEL_1-524_ IgM-ELISA.**

| **Sera** |  | **No.** | **rGroEL_1-524_**  **IgM-ELISA** | | **Panbio IgM-ELISA** | **Virion-Serion IgG-ELISA** | **Possible reason of false-positive result** |
| --- | --- | --- | --- | --- | --- | --- | --- |
|  |  |  | **1:100** | **1:1,000** |  |  |  |
| Scrub Typhus |  | SC6  SC11 | 0.83  0.34 | 0.23  0.12 | Positive | Negative | -Antibody cross-reactivity^1^  -Co-infection  -Recent *Leptospira* exposure |
|  |  | SC7  SC13  Sc23 | 0.17  1.07  1.07 | 0.05  0.04  0.31 | Negative | Negative | -Antigen cross-reactivity ^2^  -Endemic pre-existing antibody ^3^ |
| Influenza |  | IN7  IN8 | 0.16  0.2 | 0.13  0.15 | Negative | Negative | -Non-specific reaction |
| Malaria |  | MA5 | 0.15 | 0.24 | Negative | Negative | -Antigen cross-reactivity  -Endemic pre-existing antibody  -Non-specific reaction |
|  |  | MA10  MA20 | 0.2  0.15 | 0  0.16 | Negative | Positive  Positive (Borderline) | -Antibody cross-reactivity  -Recent *Leptospira* exposure |
| Melioidosis |  | ME4 | 0.32 | 0.19 | Negative | Positive | -Antibody cross-reactivity  -Recent *Leptospira* exposure |
| Dengue |  | D19A | 0.25 | Nd. | Positive | Negative | -Antibody cross-reactivity  -Recent *Leptospira* exposure  -Co-infection |
|  |  | D191A  D193A  D193C | 0.16  0.19  0.23 | Nd. | Negative | Negative | -Endemic pre-existing antibody |

Sera were laboratory-confirmed other infectious diseases. Result of rGroEL_1-524_ IgM-ELISA is expressed as optical density (OD410/650nm) .

Nd., Not determined.

^1^ Antibody cross-reactivity is defined as IgM reactivity to rGroEL_1-524_ antigen among scrub typhus, melioidosis, dengue, malaria control sera caused by previous *Leptospira* exposure or infection or co-infection.

^2^ Antigen cross reactivity is defined as sera with cross-reactivity to ortholog GroEL antigens.

^3^ Endemic pre-existing (background) antibody is defined as pre-existing anti-*Leptospira* IgG antibodies from previous exposure of patients who live in endemic leptospirosis areas.

**Supplementary Table S5.** STARD 2015 checklist for reporting diagnostic accuracy study.

| **Section and Topic** | **No** | **The STARD 2015 list** | **Page no.** |
| --- | --- | --- | --- |
| **Title or abstract** | 1 | Identification as a study of diagnostic accuracy using at least one measure of accuracy (such as sensitivity, specificity, predictive values or AUC) | 1-3 |
| **Abstract** | 2 | Structured summary of study design, methods, results and conclusions | 2-3 |
| **Introduction** | 3 | Scientific and clinical background, including the intended use and clinical role of the index test | 4-7 |
|  | 4 | Study objectives and hypothesizes | 7 |
| **Methods** |  |  |  |
| Study design | 5 | Whether data collection was planned before the index test and reference standard were performed (prospective study) or after (retrospective study) | 7-8 |
| Participants | 6 | Eligible criteria | 7-9 |
|  | 7 | On what basis potentially eligible participants were identified (such as, symptoms, results from previous test, inclusion in registry) | 8-10 |
|  | 8 | Where and when potentially eligible participants were identified (setting, location and dates) | 8-10 |
|  | 9 | Whether participants formed a consecutive, random or convenience series | 8-10 |
| Test methods | 10a | Index test, in sufficient detail to allow replication | 12-14 |
|  | 10b | Reference standard, in sufficient detail to allow replication | 8 |
|  | 11 | Rationale for choosing the reference standard (if alternatives exist) | N/A |
|  | 12a | Definition of and rationale for test positivity cut-offs or result categories of the index test, distinguishing prespecified from exploratory | 12-14 |
|  | 12b | Definition of and rationale for test positivity cut-offs or result categories of reference standard, distinguishing prespecified from exploratory | 8 |
|  | 13a | Whether clinical information and reference standard results were available to the performers or readers of the index test | 8-10, Fig. 1 |
|  | 13b | Whether clinical information and index test results were available to the assessors of the reference standard | Table 1,2  S2 Table, S3 Table |
| Analysis | 14 | Methods for estimating or comparing measures of diagnostic accuracy | 15-16 |
|  | 15 | How undetermined index test or reference standard results were handled | N/A |
|  | 16 | How missing data on the index test and reference standard were handled | Fig 1 |
|  | 17 | Any analyses of variability in diagnostic accuracy, distinguishing prespecified from exploratory | N/A |
|  | 18 | Intended sample size and how it was determined | 15 |
| **Results** |  |  |  |
| Participants | 19 | Flow of participants, using diagram | Fig 1 |
|  | 20 | Baseline demographic and clinical characteristics of participants | 8-10, Fig 1 |
|  | 21a | Distribution of severity of disease in those with the target condition | 8-10 |
|  | 21b | Distribution of alternative diagnoses in those without the target condition | N/A |
|  | 22 | Time interval and any clinical interventions between index test and reference standard | N/A |
| Test results | 23 | Cross-tabulation of index test results (or their distribution) by the results of the reference standard | Table1,2 |
|  | 24 | Estimates of diagnostic accuracy and their precision (such as 95%CI) | Table1,2 |
|  | 25 | Any adverse events from performing the index test or the reference standard | None. |
| **Discussion** | 26 | Study limitations, including sources of potential bias, statistical uncertainty and generalizability | 24-29 |
|  | 27 | Implications for practice, including the intended use and clinical role of the index test | 24-29 |
| **Other information** | 28 | Registration number and name of registry | N/A |
|  | 29 | Where the full study protocol can be accessed | Contact the authors |
|  | 30 | Sources of funding and other support; role of funders | Financial support by Center of Excellence on Medical Biotechnology (CEMB), S&T PERDO, Office of Higher Education Commission, Thailand and FTM research grant, Faculty of Tropical Medicine, Mahidol University. |
